# Bis(2,4,6-tri­amino-1,3,5-triazin-1-ium) 2-[bis­(carboxyl­atometh­yl)aza­nium­yl]acetate trihydrate

**DOI:** 10.1107/S1600536813028250

**Published:** 2013-10-19

**Authors:** Kreshnik Hoxha, Timothy J. Prior

**Affiliations:** a Department of Chemistry, University of Hull, Kingston upon Hull HU6 7RX, England

## Abstract

The title compound, 2C_3_H_7_N_6_^+^·C_6_H_7_NO_6_^2−^·3H_2_O, was obtained by mixing melamine and nitrilo­tri­acetic acid in aqueous solution. There is proton transfer from the nitrilo­triacteic acid to melamine to produce two melaminium cations and an inter­nal proton transfer to generate the [HN(CH_2_COO)]^2−^ zwitterion. The melaminium cations are arranged in hydrogen-bonded tapes formed by N—H⋯N inter­actions. These tapes extend parallel to the [010] direction and are stacked parallel to the *a* axis at a mean separation of 3.3559 (11) Å. Between these tapes lie the anions and lattice water mol­ecules. Further O—H⋯O and N—H⋯O hydrogen bonds exist between the water mol­ecules, the anions, and the melaminium cations, generating a three-dimensional array. The crystal examined was found to be twinned by a twofold rotation about the direct lattice direction [100]. The two twin components were present in the ratio 0.5918:0.4082 (14).

## Related literature

For compounds of melamine with simple carb­oxy­lic acid, see, for example: Froschauer & Weil (2012 ▶); Eppel & Bernstein (2009 ▶); Perpétuo & Janczak (2002 ▶). For those with tri­carb­oxy­lic acids, see: Eshtiagh-Hosseini *et al.* (2010 ▶); Huczynski *et al.* (2009 ▶); Perpetuo & Janczak (2003 ▶). For assignment of protonation on the grounds of bond angle and bond length, see: Childs *et al.* (2007 ▶) and Hingerty *et al.* (1981 ▶), respectively. An introduction to graph-set theory may be found in Etter *et al.* (1990 ▶). 
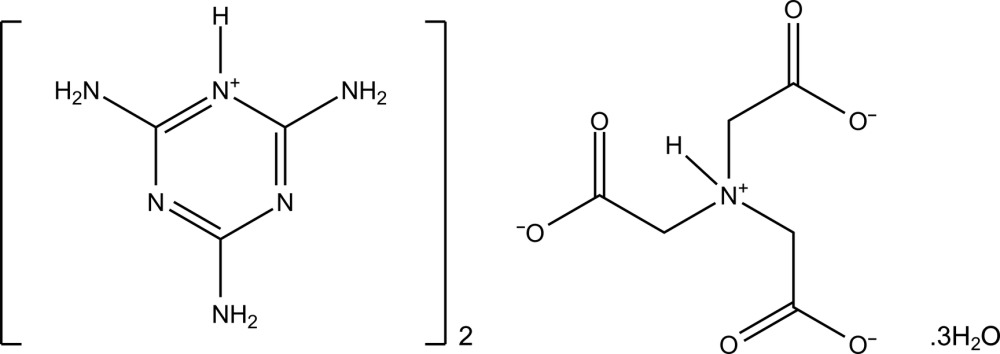


## Experimental

### 

#### Crystal data

2C_3_H_7_N_6_^+^·C_6_H_7_NO_6_^2−^·3H_2_O
*M* *_r_* = 497.43
Triclinic, 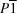

*a* = 6.7117 (11) Å
*b* = 12.1495 (19) Å
*c* = 13.102 (3) Å
α = 82.714 (15)°
β = 89.252 (16)°
γ = 83.238 (13)°
*V* = 1052.4 (3) Å^3^
*Z* = 2
Mo *K*α radiation
μ = 0.13 mm^−1^
*T* = 150 K
0.36 × 0.16 × 0.04 mm

#### Data collection

Stoe IPDS2 diffractometer
Absorption correction: analytical (*X-RED* and *X-SHAPE*; Stoe & Cie, 2002 ▶) *T*_min_ = 0.957, *T*_max_ = 0.994
10946 measured reflections
10946 independent reflections
5515 reflections with *I* > 2σ(*I*)

#### Refinement

*R*[*F* ^2^ > 2σ(*F* ^2^)] = 0.084
*wR*(*F* ^2^) = 0.244
*S* = 0.95
10946 reflections
327 parameters
10 restraints
H atoms treated by a mixture of independent and constrained refinement
Δρ_max_ = 0.40 e Å^−3^
Δρ_min_ = −0.48 e Å^−3^

### 

Data collection: *X-AREA* (Stoe & Cie, 2002 ▶); cell refinement: *X-AREA*; data reduction: *X-RED* (Stoe & Cie, 2002 ▶); program(s) used to solve structure: *SHELXS86* (Sheldrick, 2008 ▶); program(s) used to refine structure: *SHELXL97* (Sheldrick, 2008 ▶); molecular graphics: *SHELXTL* (Sheldrick, 2008 ▶); software used to prepare material for publication: *SHELXL97*.

## Supplementary Material

Crystal structure: contains datablock(s) I, New_Global_Publ_Block. DOI: 10.1107/S1600536813028250/zl2565sup1.cif

Structure factors: contains datablock(s) I. DOI: 10.1107/S1600536813028250/zl2565Isup2.hkl

Supplementary material file. DOI: 10.1107/S1600536813028250/zl2565Isup3.cdx

Supplementary material file. DOI: 10.1107/S1600536813028250/zl2565Isup4.cml

Additional supplementary materials:  crystallographic information; 3D view; checkCIF report

## Figures and Tables

**Table 1:** Hydrogen-bond geometry (Å, °)

*D*—H⋯*A*	*D*—H	H⋯*A*	*D*⋯*A*	*D*—H⋯*A*
N3—H3⋯O5	0.88	2.60	3.296 (4)	136
N3—H3⋯O6	0.88	1.82	2.684 (4)	166
N11—H11*A*⋯O3^i^	0.88	2.18	3.042 (4)	166
N11—H11*B*⋯O1*W* ^ii^	0.88	2.13	2.989 (4)	164
N12—H12*A*⋯N21	0.88	2.04	2.915 (4)	174
N12—H12*B*⋯O2^iii^	0.88	2.25	2.904 (4)	131
N12—H12*B*⋯O6	0.88	2.58	3.260 (4)	135
N13—H13*A*⋯N22^iv^	0.88	2.13	3.012 (4)	176
N13—H13*B*⋯O5	0.88	2.03	2.870 (4)	159
N23—H23⋯O2*W* ^v^	0.88	1.94	2.793 (4)	164
N31—H31*A*⋯O3*W* ^vi^	0.88	2.06	2.870 (4)	153
N31—H31*B*⋯O2^iii^	0.88	2.22	3.048 (4)	157
N32—H32*A*⋯N1^vii^	0.88	2.06	2.933 (4)	173
N32—H32*B*⋯O3^viii^	0.88	2.11	2.817 (4)	137
N33—H33*A*⋯N2	0.88	2.09	2.973 (4)	177
N33—H33*B*⋯O1*W* ^ii^	0.88	2.19	2.850 (4)	132
N50—H50⋯O1^iii^	0.93	2.28	2.969 (4)	130
C55—H55*A*⋯O4^ix^	0.99	2.55	3.470 (5)	154
O1*W*—H1*AW*⋯O1^x^	0.84 (2)	2.02 (2)	2.856 (4)	172 (4)
O1*W*—H1*BW*⋯O2	0.84 (2)	1.98 (3)	2.775 (3)	157 (4)
O2*W*—H2*AW*⋯O3^xi^	0.83 (2)	2.57 (3)	3.265 (4)	143 (4)
O2*W*—H2*AW*⋯O4^xi^	0.83 (2)	2.38 (3)	3.168 (4)	159 (4)
O2*W*—H2*BW*⋯O4	0.81 (2)	2.08 (2)	2.812 (4)	150 (4)
O3*W*—H3*AW*⋯O4	0.84 (2)	1.95 (3)	2.768 (4)	163 (5)
O3*W*—H3*BW*⋯O5^xi^	0.83 (2)	1.96 (3)	2.773 (4)	165 (5)

Symmetry codes: (i) 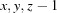; (ii) 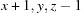; (iii) 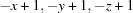; (iv) 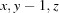; (v) 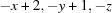; (vi) 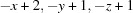; (vii) 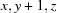; (viii) 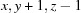; (ix) 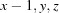; (x) 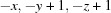; (xi) 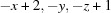.

**Fig. 1.**
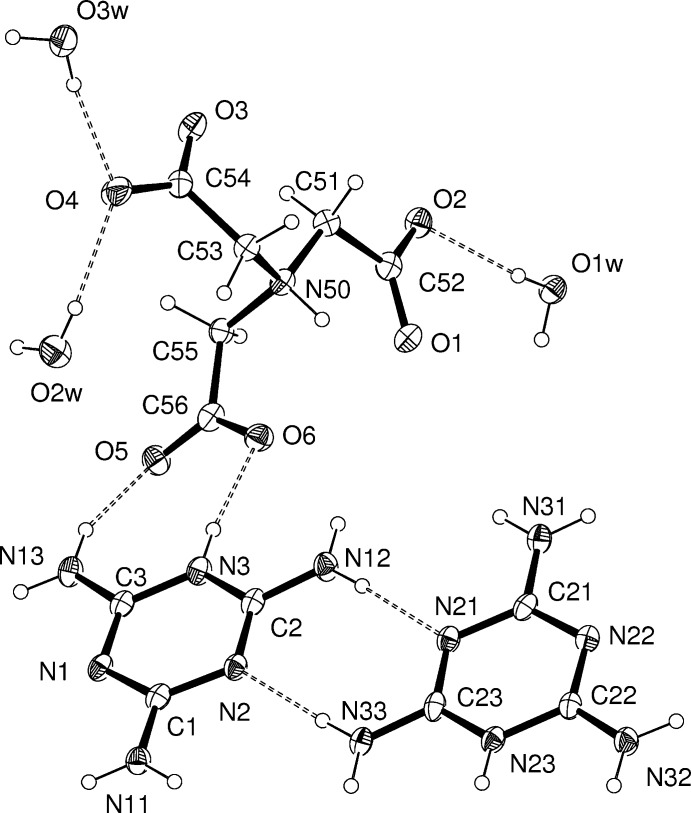
ORTEP representation of the assymetric unit of 1 with atoms drawn as 50% thermal ellipsoids. Dashed lines indicate hydrogen bonds.

**Fig. 2.**
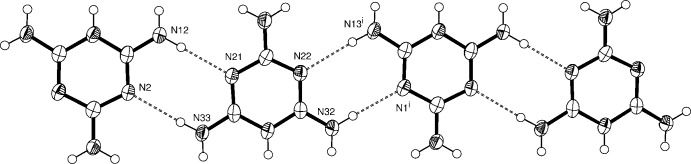
Single hydrogen-bonded tape formed from melaminium cations. Hydrogen bonds are represented as dashed lines. Symmetry equivalent atoms are generated by i = x, 1+y, z..

**Fig. 3.**
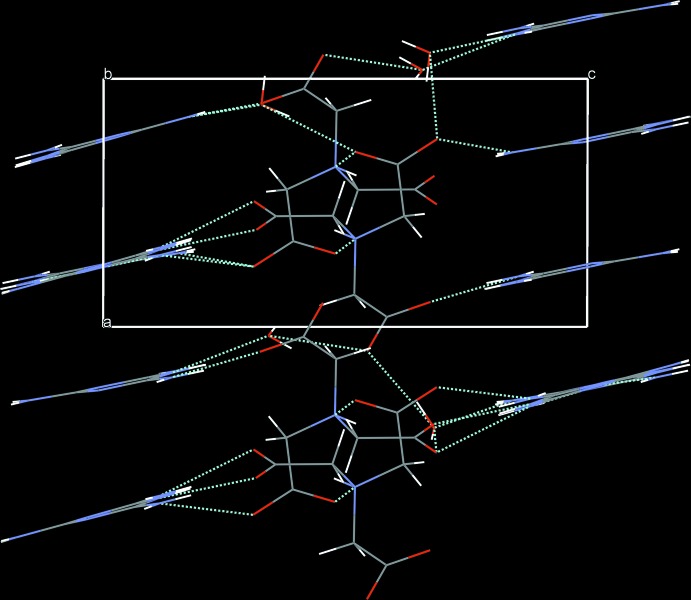
Crystal packing within 1 viewed down [010]. Stacks of hydrogen-bonded tapes are linked by hydrogen bonds to the anion and water. Dashed lines indicate hydrogen bonds.

## supplementary crystallographic information

### 1. Comment

Melamine is an organic base with a pronounced tendency to form hydrogen-bonded architectures in combination with carboxylic acids. Many of these compounds display common structural features and normally these are best classed as salts with full proton transfer to form melaminium cations. These melaminium cations then form hydrogen-bonded tapes composed of N–H···N hydrogen bonds. The tapes have a propensity to assemble into π-stacks and between these stacks are located the carboxylates. Several examples with simple alkyl carboxylic acids (see for example Perpétuo & Janczak, 2002) and dicarboxylic acids are known (such as Froschauer and Weil, 2012 & Eppel and Bernstein, 2009). Those with three carboxylic acid groups are less common (Huczynski *et al.*, 2009; Eshtiagh-Hosseini *et al.*, 2010; Perpetuo and Janczak, 2003). We set out to explore the effect of introducing multiple carboxylic acid groups with a flexible geometry through the use of nitrilotriacetic acid, N(CH_2_COOH)_3_.

The title compound, 1, crystallizes in the triclinic space group *P*1. The asymmetric unit (illustrated in Figure 1) contains two crystallographically independent melaminium cations, the nitrilotriacetate anion and three water molecules. The crystal examined was found to be twinned by a 2-fold rotation about [100] corresponding to the twin law (1 0 0 0.422 -1 0 0.051 0 -1). It was possible to index the diffraction images using two twin domains. The integration procedure employed both twin domains. The structure was refined using all observed data using the HKLF5 formalism within *SHELXL97*. The relative amount of the two components was refined to be 0.5918: 0.4082 (14).

The assignment of hydrogen bonding between different components was greatly facilitated by the identification of hydrogen atoms with final difference Fourier maps. Examination of hydrogen locations reveals that crystallization of this compound is associated with proton migration from acid to the base. All three of the carboxylic acid groups are deprotonated. Two carboxylic protons migrate to one of the endocyclic N atoms in each melamine moiety giving rise to two melaminium cations. The third carboxylic acid proton migrates to the central nitrogen in the nitrilotriacetic acid moiety to generate an anion correctly called 2,2',2''-ammoniotriacetate. As in similar cases full proton transfer means these compounds are best described as salts rather than co-crystals. The assignment of the carboxylates is in line with work of Childs *et al.* (2007) who noted that a similarity in C—O bond lengths (ΔD_C—O_ < 0.03 Å) is indicative of carboxylate rather than carboxylic acid. For the three COO groups in the anion, the mean C—O bond length is 1.253 (3) Å, but the deviation from equal values for the three pairs of C–O distances are 0.02, 0.008, and 0.003 Å. Similarly, examination of the bond angles in the endocyclic protonated nitrogen in both melamine moieties reveals that there is a marked bond angle increase in the aromatic ring at the protonated nitrogen compared to the unprotonated endocyclic nitrogen atoms. This agrees with the work of Hingerty *et al.* (1981) who reported an increase in bond angle at the endocyclic nitrogen atoms upon protonation. At the protonated N atoms the bond angles are 119.1 (2) and 119.3 (2) ° compared with the other C—N—C bond angles which have a mean of 115.8 (2) °.

The two melaminium cations form hydrogen bonded tapes sustained by N—H···N interactions, common to many other similar compounds. Pairs of N—H···N interactions between adjacent melaminium cations form embraces with graph set notation *R*^2^_2_(8) (Etter *et al.*, 1990). There are two symmetry independent embraces of this type which assemble the melaminium cations into tapes that extend parallel to the crystallographic [010] direction. These tapes are stacked parallel to the *a* axis at a mean separation of *a*/2 (3.3559 (11) Å) suggestive of π-stacking. A single tape is illustrated in Figure 2. Between the tapes lie the [HN(CH_2_COO)_3_]^2-^ anions and water molecules. Each carboxylate acts as a hydrogen bond acceptor to amino groups of the melaminium cations. In this way the anions are involved in N—H···O hydrogen bonds between different tapes within the same stack and between different stacks. Additional N—H···O and O—H···O hydrogen bonds formed by the water molecules present further help to knit together the cations and anions. Full details of the hydrogen bonds present are contained within Table 1. The crystal packing within 1 is represented in Figure 3.

The crystal structures of melamine with monocarboxylic acids form a well established family of compounds. Similarly linear dicarboxylic acid salts of melamine are well illustrated. These have similar structural features (the π-stacking of tapes of melaminium held together by *R*^2^_2_(8) embraces). The structure of 1 shows analogous features to those of simpler carboxylates.

### 2. Experimental

Melamine (0.334 g, 2 mmol) and nitrilotriacetic acid (0.249 g,1 mmol) were dissolved in 50:50 ethanol:water (20 ml) and stirred for 15 min. The solution was left unperturbed for slow solvent evaporation in suitably sized vials. After approximately 3 days, colourless needle-shaped crystals were obtained.

### 3. Refinement

The crystal examined was found to be twinned by a 2-fold rotation about [100]. The twinning was apparent in the diffraction images. The twin law was identified by inspection of reciprocal space within *X-AREA* (Stoe & Cie, 2002). It was possible to index the diffraction images using two twin domains. The integration procedure employed both twin domains. The structure was refined using all observed data using the HKLF5 formalism within *SHELXL97*. The relative amount of the two components was refined to be 0.5918: 0.4082 (14). Refinement using a single twin domain was not satisfactory as approximately 40% of the spots from the first domain were overlapped by those from the second domain.

Hydrogen atoms were located in difference Fourier maps. Those on the organic components were placed with a riding model with *U*_iso_ set to 1.2 times the *U*_eq_ of the atom on which they ride. Hydrogen atoms attached to water were refined subject to sensible bond length and bond angle restraints with *U*_iso_ values set at 1.5 times the *U*_eq_ of the central oxygen atom.

### Crystal data

**Table:**

2C_3_H_7_N_6_^+^·C_6_H_7_NO_6_^2^^−^·3H_2_O	*Z* = 2
*M**_r_* = 497.43	*F*(000) = 524
Triclinic, *P*1	*D*_x_ = 1.570 Mg m^−^^3^
Hall symbol: -P 1	Mo *K*α radiation, λ = 0.71073 Å
*a* = 6.7117 (11) Å	Cell parameters from 4000 reflections
*b* = 12.1495 (19) Å	θ = 1.7–29.5°
*c* = 13.102 (3) Å	µ = 0.13 mm^−^^1^
α = 82.714 (15)°	*T* = 150 K
β = 89.252 (16)°	Needle, colourless
γ = 83.238 (13)°	0.36 × 0.16 × 0.04 mm
*V* = 1052.4 (3) Å^3^	

### Data collection

**Table:**

Stoe IPDS2 diffractometer	10946 independent reflections
Radiation source: fine-focus sealed tube	5515 reflections with *I* > 2σ(*I*)
Graphite monochromator	*R*_int_ = 0.000
Detector resolution: 6.67 pixels mm^-1^	θ_max_ = 25.3°, θ_min_ = 1.7°
ω scans	*h* = −8→7
Absorption correction: analytical (*X-RED* and *X-SHAPE*; Stoe & Cie, 2002)	*k* = −14→14
*T*_min_ = 0.957, *T*_max_ = 0.994	*l* = −15→15
10946 measured reflections	

### Refinement

**Table:**

Refinement on *F*^2^	Primary atom site location: structure-invariant direct methods
Least-squares matrix: full	Secondary atom site location: difference Fourier map
*R*[*F*^2^ > 2σ(*F*^2^)] = 0.084	Hydrogen site location: inferred from neighbouring sites
*wR*(*F*^2^) = 0.244	H atoms treated by a mixture of independent and constrained refinement
*S* = 0.95	*w* = 1/[σ^2^(*F*_o_^2^) + (0.1396*P*)^2^] where *P* = (*F*_o_^2^ + 2*F*_c_^2^)/3
10946 reflections	(Δ/σ)_max_ < 0.001
327 parameters	Δρ_max_ = 0.40 e Å^−^^3^
10 restraints	Δρ_min_ = −0.48 e Å^−^^3^

### Special details

**Table:**

Experimental. a face indexed abosorption correction was applied. this utilised the Tompa method implmented within Stoe X-Area.
Geometry. All e.s.d.'s (except the e.s.d. in the dihedral angle between two l.s. planes) are estimated using the full covariance matrix. The cell e.s.d.'s are taken into account individually in the estimation of e.s.d.'s in distances, angles and torsion angles; correlations between e.s.d.'s in cell parameters are only used when they are defined by crystal symmetry. An approximate (isotropic) treatment of cell e.s.d.'s is used for estimating e.s.d.'s involving l.s. planes.
Refinement. Refinement of *F*^2^ against ALL reflections. The weighted *R*-factor *wR* and goodness of fit *S* are based on *F*^2^, conventional *R*-factors *R* are based on *F*, with *F* set to zero for negative *F*^2^. The threshold expression of *F*^2^ > σ(*F*^2^) is used only for calculating *R*-factors(gt) *etc*. and is not relevant to the choice of reflections for refinement. *R*-factors based on *F*^2^ are statistically about twice as large as those based on *F*, and *R*- factors based on ALL data will be even larger.

### Fractional atomic coordinates and isotropic or equivalent isotropic displacement parameters (Å^2^)

**Table:**

	*x*	*y*	*z*	*U*_iso_*/*U*_eq_	
C1	0.7947 (5)	0.3396 (3)	−0.0788 (3)	0.0284 (8)	
C2	0.7256 (5)	0.4231 (3)	0.0654 (3)	0.0282 (8)	
C3	0.7125 (5)	0.2304 (3)	0.0665 (3)	0.0269 (8)	
N1	0.7670 (4)	0.2371 (2)	−0.0319 (2)	0.0288 (7)	
N2	0.7753 (4)	0.4355 (2)	−0.0339 (2)	0.0283 (7)	
N3	0.6891 (4)	0.3237 (2)	0.1167 (2)	0.0308 (7)	
H3	0.6508	0.3189	0.1815	0.037*	
N11	0.8471 (4)	0.3485 (2)	−0.1770 (2)	0.0312 (7)	
H11A	0.8626	0.2887	−0.2093	0.037*	
H11B	0.8665	0.4140	−0.2101	0.037*	
N12	0.7108 (4)	0.5111 (2)	0.1170 (2)	0.0330 (7)	
H12A	0.7337	0.5768	0.0856	0.040*	
H12B	0.6781	0.5037	0.1826	0.040*	
N13	0.6798 (4)	0.1346 (2)	0.1184 (3)	0.0329 (7)	
H13A	0.6939	0.0735	0.0882	0.039*	
H13B	0.6438	0.1314	0.1834	0.039*	
C21	0.7361 (5)	0.8238 (3)	0.0554 (3)	0.0282 (8)	
C22	0.7847 (5)	0.9412 (3)	−0.0907 (3)	0.0284 (8)	
C23	0.7972 (5)	0.7470 (3)	−0.0937 (3)	0.0295 (8)	
N21	0.7571 (4)	0.7302 (2)	0.0062 (2)	0.0283 (7)	
N22	0.7439 (4)	0.9294 (2)	0.0094 (2)	0.0298 (7)	
N23	0.8157 (4)	0.8499 (2)	−0.1437 (2)	0.0293 (7)	
H23	0.8472	0.8584	−0.2094	0.035*	
N31	0.7031 (4)	0.8087 (2)	0.1552 (2)	0.0337 (7)	
H31A	0.6869	0.8664	0.1901	0.040*	
H31B	0.6974	0.7409	0.1870	0.040*	
N32	0.7965 (5)	1.0404 (2)	−0.1423 (2)	0.0348 (7)	
H32A	0.7773	1.1004	−0.1106	0.042*	
H32B	0.8237	1.0467	−0.2085	0.042*	
N33	0.8209 (4)	0.6595 (2)	−0.1465 (2)	0.0309 (7)	
H33A	0.8100	0.5920	−0.1150	0.037*	
H33B	0.8475	0.6691	−0.2127	0.037*	
N50	0.6450 (4)	0.3141 (2)	0.5208 (2)	0.0274 (6)	
H50	0.6099	0.3799	0.4771	0.033*	
C51	0.5536 (5)	0.3342 (3)	0.6213 (3)	0.0312 (8)	
H51A	0.6403	0.3772	0.6577	0.037*	
H51B	0.5447	0.2618	0.6641	0.037*	
C52	0.3453 (5)	0.3985 (3)	0.6077 (3)	0.0310 (8)	
C53	0.8705 (5)	0.2958 (3)	0.5187 (3)	0.0293 (8)	
H53A	0.9235	0.3612	0.5418	0.035*	
H53B	0.9149	0.2911	0.4469	0.035*	
C54	0.9599 (5)	0.1904 (3)	0.5862 (3)	0.0306 (8)	
C55	0.5523 (5)	0.2261 (3)	0.4741 (3)	0.0289 (8)	
H55A	0.4121	0.2248	0.4983	0.035*	
H55B	0.6271	0.1521	0.4970	0.035*	
C56	0.5540 (5)	0.2475 (3)	0.3566 (3)	0.0321 (8)	
O1	0.2949 (4)	0.4461 (2)	0.5204 (2)	0.0343 (6)	
O2	0.2429 (4)	0.4017 (2)	0.6894 (2)	0.0354 (6)	
O3	0.8994 (4)	0.1721 (2)	0.6763 (2)	0.0393 (7)	
O4	1.0971 (4)	0.1308 (2)	0.5460 (2)	0.0509 (8)	
O5	0.4934 (4)	0.1730 (2)	0.3111 (2)	0.0375 (6)	
O6	0.6089 (4)	0.3375 (2)	0.3162 (2)	0.0366 (6)	
O1W	−0.1047 (4)	0.5512 (2)	0.6739 (2)	0.0350 (6)	
H1AW	−0.152 (6)	0.546 (4)	0.616 (2)	0.053*	
H1BW	0.012 (4)	0.520 (3)	0.667 (3)	0.053*	
O2W	1.0355 (5)	0.0954 (2)	0.3413 (2)	0.0458 (7)	
H2AW	1.002 (8)	0.032 (2)	0.355 (4)	0.069*	
H2BW	1.087 (7)	0.117 (4)	0.3894 (16)	0.069*	
O3W	1.4067 (5)	0.0556 (2)	0.6843 (2)	0.0460 (7)	
H3AW	1.302 (5)	0.067 (4)	0.648 (4)	0.069*	
H3BW	1.455 (7)	−0.010 (2)	0.679 (4)	0.069*	

### Atomic displacement parameters (Å^2^)

**Table:**

	*U*^11^	*U*^22^	*U*^33^	*U*^12^	*U*^13^	*U*^23^
C1	0.0177 (17)	0.0277 (19)	0.039 (2)	−0.0022 (13)	−0.0022 (15)	−0.0002 (16)
C2	0.0198 (17)	0.0231 (18)	0.041 (2)	−0.0033 (13)	−0.0019 (15)	−0.0003 (15)
C3	0.0167 (16)	0.0252 (18)	0.038 (2)	−0.0027 (13)	−0.0014 (15)	−0.0003 (16)
N1	0.0241 (15)	0.0238 (15)	0.0374 (18)	−0.0041 (12)	−0.0017 (13)	0.0018 (13)
N2	0.0287 (15)	0.0220 (15)	0.0340 (17)	−0.0041 (12)	−0.0011 (13)	−0.0011 (13)
N3	0.0306 (16)	0.0251 (15)	0.0363 (18)	−0.0063 (12)	0.0025 (13)	0.0000 (13)
N11	0.0342 (16)	0.0253 (15)	0.0340 (18)	−0.0061 (12)	0.0023 (13)	−0.0009 (13)
N12	0.0413 (18)	0.0236 (15)	0.0348 (18)	−0.0078 (12)	0.0050 (14)	−0.0033 (13)
N13	0.0350 (17)	0.0230 (15)	0.0407 (19)	−0.0083 (12)	0.0041 (14)	0.0005 (14)
C21	0.0200 (17)	0.0247 (18)	0.038 (2)	−0.0021 (13)	−0.0007 (15)	0.0027 (16)
C22	0.0236 (18)	0.0228 (18)	0.039 (2)	−0.0023 (13)	−0.0027 (15)	−0.0047 (16)
C23	0.0191 (17)	0.0218 (18)	0.047 (2)	−0.0013 (13)	−0.0018 (15)	−0.0051 (16)
N21	0.0263 (15)	0.0221 (15)	0.0360 (18)	−0.0036 (11)	0.0008 (13)	−0.0011 (13)
N22	0.0281 (15)	0.0254 (16)	0.0353 (18)	−0.0023 (12)	0.0002 (13)	−0.0027 (13)
N23	0.0297 (16)	0.0222 (15)	0.0354 (17)	−0.0043 (12)	−0.0018 (13)	0.0009 (13)
N31	0.0356 (17)	0.0254 (16)	0.0396 (19)	−0.0038 (13)	0.0031 (14)	−0.0029 (14)
N32	0.0462 (18)	0.0241 (16)	0.0337 (18)	−0.0043 (13)	0.0028 (15)	−0.0021 (14)
N33	0.0326 (16)	0.0215 (15)	0.0373 (18)	−0.0020 (12)	0.0022 (13)	0.0000 (13)
N50	0.0253 (15)	0.0250 (15)	0.0314 (16)	−0.0067 (11)	0.0026 (12)	0.0019 (12)
C51	0.0286 (19)	0.0328 (19)	0.032 (2)	−0.0057 (14)	0.0007 (15)	−0.0028 (16)
C52	0.0275 (18)	0.0283 (19)	0.038 (2)	−0.0097 (14)	0.0008 (16)	−0.0031 (17)
C53	0.0252 (18)	0.0302 (19)	0.032 (2)	−0.0037 (14)	0.0039 (15)	−0.0006 (16)
C54	0.0225 (18)	0.0301 (19)	0.040 (2)	−0.0041 (14)	−0.0017 (16)	−0.0066 (17)
C55	0.0293 (18)	0.0261 (18)	0.031 (2)	−0.0056 (14)	0.0010 (15)	−0.0025 (16)
C56	0.0263 (19)	0.029 (2)	0.040 (2)	−0.0057 (15)	−0.0002 (16)	0.0005 (17)
O1	0.0307 (13)	0.0319 (14)	0.0381 (16)	−0.0018 (10)	−0.0008 (11)	0.0028 (12)
O2	0.0302 (14)	0.0356 (14)	0.0395 (15)	−0.0013 (10)	0.0061 (12)	−0.0041 (12)
O3	0.0345 (15)	0.0348 (15)	0.0441 (17)	0.0033 (11)	0.0022 (13)	0.0049 (13)
O4	0.0479 (17)	0.0594 (19)	0.0383 (16)	0.0229 (14)	0.0003 (14)	−0.0058 (14)
O5	0.0387 (15)	0.0350 (14)	0.0406 (15)	−0.0095 (11)	0.0011 (12)	−0.0068 (12)
O6	0.0425 (15)	0.0343 (14)	0.0331 (15)	−0.0087 (12)	−0.0005 (12)	−0.0001 (12)
O1W	0.0300 (14)	0.0362 (15)	0.0379 (15)	−0.0015 (11)	0.0013 (12)	−0.0033 (12)
O2W	0.0554 (18)	0.0409 (17)	0.0435 (17)	−0.0172 (14)	0.0046 (14)	−0.0040 (14)
O3W	0.0483 (18)	0.0360 (15)	0.0522 (19)	0.0020 (13)	−0.0064 (14)	−0.0056 (14)

### Geometric parameters (Å, º)

**Table:**

C1—N11	1.324 (5)	N32—H32B	0.8800
C1—N1	1.350 (4)	N33—H33A	0.8800
C1—N2	1.362 (4)	N33—H33B	0.8800
C2—N12	1.329 (4)	N50—C51	1.482 (4)
C2—N2	1.334 (5)	N50—C55	1.496 (4)
C2—N3	1.353 (4)	N50—C53	1.504 (5)
C3—N13	1.312 (4)	N50—H50	0.9300
C3—N1	1.330 (5)	C51—C52	1.520 (5)
C3—N3	1.373 (4)	C51—H51A	0.9900
N3—H3	0.8800	C51—H51B	0.9900
N11—H11A	0.8800	C52—O1	1.247 (5)
N11—H11B	0.8800	C52—O2	1.267 (4)
N12—H12A	0.8800	C53—C54	1.525 (5)
N12—H12B	0.8800	C53—H53A	0.9900
N13—H13A	0.8800	C53—H53B	0.9900
N13—H13B	0.8800	C54—O3	1.246 (5)
C21—N31	1.317 (5)	C54—O4	1.256 (4)
C21—N22	1.353 (4)	C55—C56	1.528 (5)
C21—N21	1.368 (4)	C55—H55A	0.9900
C22—N32	1.315 (4)	C55—H55B	0.9900
C22—N22	1.329 (5)	C56—O6	1.247 (4)
C22—N23	1.375 (4)	C56—O5	1.253 (4)
C23—N21	1.328 (5)	O1W—H1AW	0.84 (2)
C23—N33	1.333 (4)	O1W—H1BW	0.84 (2)
C23—N23	1.355 (4)	O2W—H2AW	0.83 (2)
N23—H23	0.8800	O2W—H2BW	0.81 (2)
N31—H31A	0.8800	O3W—H3AW	0.84 (2)
N31—H31B	0.8800	O3W—H3BW	0.83 (2)
N32—H32A	0.8800		
			
N11—C1—N1	117.6 (3)	C22—N32—H32B	120.0
N11—C1—N2	116.6 (3)	H32A—N32—H32B	120.0
N1—C1—N2	125.8 (3)	C23—N33—H33A	120.0
N12—C2—N2	119.4 (3)	C23—N33—H33B	120.0
N12—C2—N3	118.1 (3)	H33A—N33—H33B	120.0
N2—C2—N3	122.5 (3)	C51—N50—C55	112.0 (3)
N13—C3—N1	121.0 (3)	C51—N50—C53	115.8 (3)
N13—C3—N3	118.1 (3)	C55—N50—C53	112.2 (3)
N1—C3—N3	120.9 (3)	C51—N50—H50	105.2
C3—N1—C1	116.5 (3)	C55—N50—H50	105.2
C2—N2—C1	115.0 (3)	C53—N50—H50	105.2
C2—N3—C3	119.2 (3)	N50—C51—C52	111.3 (3)
C2—N3—H3	120.4	N50—C51—H51A	109.4
C3—N3—H3	120.4	C52—C51—H51A	109.4
C1—N11—H11A	120.0	N50—C51—H51B	109.4
C1—N11—H11B	120.0	C52—C51—H51B	109.4
H11A—N11—H11B	120.0	H51A—C51—H51B	108.0
C2—N12—H12A	120.0	O1—C52—O2	126.5 (3)
C2—N12—H12B	120.0	O1—C52—C51	118.3 (3)
H12A—N12—H12B	120.0	O2—C52—C51	115.1 (3)
C3—N13—H13A	120.0	N50—C53—C54	113.7 (3)
C3—N13—H13B	120.0	N50—C53—H53A	108.8
H13A—N13—H13B	120.0	C54—C53—H53A	108.8
N31—C21—N22	118.1 (3)	N50—C53—H53B	108.8
N31—C21—N21	116.6 (3)	C54—C53—H53B	108.8
N22—C21—N21	125.2 (3)	H53A—C53—H53B	107.7
N32—C22—N22	121.2 (3)	O3—C54—O4	125.1 (3)
N32—C22—N23	117.8 (3)	O3—C54—C53	118.9 (3)
N22—C22—N23	121.0 (3)	O4—C54—C53	115.9 (3)
N21—C23—N33	118.9 (3)	N50—C55—C56	111.5 (3)
N21—C23—N23	122.2 (3)	N50—C55—H55A	109.3
N33—C23—N23	119.0 (3)	C56—C55—H55A	109.3
C23—N21—C21	115.6 (3)	N50—C55—H55B	109.3
C22—N22—C21	116.5 (3)	C56—C55—H55B	109.3
C23—N23—C22	119.4 (3)	H55A—C55—H55B	108.0
C23—N23—H23	120.3	O6—C56—O5	126.9 (4)
C22—N23—H23	120.3	O6—C56—C55	117.4 (3)
C21—N31—H31A	120.0	O5—C56—C55	115.6 (3)
C21—N31—H31B	120.0	H1AW—O1W—H1BW	100 (4)
H31A—N31—H31B	120.0	H2AW—O2W—H2BW	113 (4)
C22—N32—H32A	120.0	H3AW—O3W—H3BW	105 (4)

### Hydrogen-bond geometry (Å, º)

**Table:**

*D*—H···*A*	*D*—H	H···*A*	*D*···*A*	*D*—H···*A*
N3—H3···O5	0.88	2.60	3.296 (4)	136
N3—H3···O6	0.88	1.82	2.684 (4)	166
N11—H11*A*···O3^i^	0.88	2.18	3.042 (4)	166
N11—H11*B*···O1*W*^ii^	0.88	2.13	2.989 (4)	164
N12—H12*A*···N21	0.88	2.04	2.915 (4)	174
N12—H12*B*···O2^iii^	0.88	2.25	2.904 (4)	131
N12—H12*B*···O6	0.88	2.58	3.260 (4)	135
N13—H13*A*···N22^iv^	0.88	2.13	3.012 (4)	176
N13—H13*B*···O5	0.88	2.03	2.870 (4)	159
N23—H23···O2*W*^v^	0.88	1.94	2.793 (4)	164
N31—H31*A*···O3*W*^vi^	0.88	2.06	2.870 (4)	153
N31—H31*B*···O2^iii^	0.88	2.22	3.048 (4)	157
N32—H32*A*···N1^vii^	0.88	2.06	2.933 (4)	173
N32—H32*B*···O3^viii^	0.88	2.11	2.817 (4)	137
N33—H33*A*···N2	0.88	2.09	2.973 (4)	177
N33—H33*B*···O1*W*^ii^	0.88	2.19	2.850 (4)	132
N50—H50···O1^iii^	0.93	2.28	2.969 (4)	130
C55—H55*A*···O4^ix^	0.99	2.55	3.470 (5)	154
O1*W*—H1*AW*···O1^x^	0.84 (2)	2.02 (2)	2.856 (4)	172 (4)
O1*W*—H1*BW*···O2	0.84 (2)	1.98 (3)	2.775 (3)	157 (4)
O2*W*—H2*AW*···O3^xi^	0.83 (2)	2.57 (3)	3.265 (4)	143 (4)
O2*W*—H2*AW*···O4^xi^	0.83 (2)	2.38 (3)	3.168 (4)	159 (4)
O2*W*—H2*BW*···O4	0.81 (2)	2.08 (2)	2.812 (4)	150 (4)
O3*W*—H3*AW*···O4	0.84 (2)	1.95 (3)	2.768 (4)	163 (5)
O3*W*—H3*BW*···O5^xi^	0.83 (2)	1.96 (3)	2.773 (4)	165 (5)

Symmetry codes: (i) *x*, *y*, *z*−1; (ii) *x*+1, *y*, *z*−1; (iii) −*x*+1, −*y*+1, −*z*+1; (iv) *x*, *y*−1, *z*; (v) −*x*+2, −*y*+1, −*z*; (vi) −*x*+2, −*y*+1, −*z*+1; (vii) *x*, *y*+1, *z*; (viii) *x*, *y*+1, *z*−1; (ix) *x*−1, *y*, *z*; (x) −*x*, −*y*+1, −*z*+1; (xi) −*x*+2, −*y*, −*z*+1.
